# Impact of sampling depth on CO$_{2}$ flux estimates

**DOI:** 10.1038/s41598-024-69177-x

**Published:** 2024-08-09

**Authors:** Cátia C. Azevedo, Melchor González-Dávila, J. Magdalena Santana-Casiano, David González-Santana, Rui M. A. Caldeira

**Affiliations:** 1 Dom Luiz Institute, Faculty of Sciences, University of Lisbon, Lisbon, Portugal; 2 Oceanic Observatory of Madeira, ARDITI, Funchal, Portugal; 3 Instituto de Oceanografía y Cambio Global, IOCAG, Universidad de Las Palmas de Gran Canaria, 35017 Las Palmas de Gran Canaria, Spain

**Keywords:** Marine chemistry, Thermodynamics, Carbon cycle, Physical oceanography

## Abstract

The exchange of trace gases between the atmosphere and the ocean plays a key role in the Earth’s climate. Fluxes at the air-sea interface are affected mainly by wind blowing over the ocean and seawater temperature and salinity changes. This study aimed to quantify the use of CO$_{2}$ partial pressure (pCO$_{2}$) measurements at different depths (1, 5, and 10 m) in ocean surface layers to determine CO$_{2}$ fluxes (FCO$_{2}$) and to investigate the impacts of wind-sheltered and wind-exposed regions on the carbon budget. Vertical profiles of temperature, salinity, and pCO$_{2}$ were considered during a daily cycle. pCO$_{2}$ profiles exhibited relatively high values during sunny hours, associated with relatively high sea temperatures. However, the largest FCO$_{2}$ corresponded with higher wind speeds. Estimated fluxes between measurements at 1 and 10 m depths decreased by 71% in the sheltered region and 44% in the exposed region. According to the SOCAT dataset, at a depth of 5 m, the Atlantic basin emits approximately 0.29 Tg month$^{-1}$ of CO$_{2}$ to the atmosphere; nevertheless, our estimates suggest that FCO$_{2}$ at the surface is 12.02 Tg month$^{-1}$, which is 97.6% greater than that at 5 m depth. Therefore, future studies should consider sampling depth to adequately estimate the FCO$_{2}$.

## Introduction

Earth’s oceans are important carbon sinks, removing an estimated 25%^1–3^ to 30%^4^ of the total CO$_{2}$ emissions from the atmosphere. Gas exchange across the air-sea interface is driven mainly by wind blowing over the sea surface^5^ and changes in seawater temperature and salinity. The latter changes influence the solubility of dissolved gases and thus the amount available for air–sea exchange^6^. Understanding the associated processes is essential for quantifying air-sea CO$_{2}$ fluxes (FCO$_{2}$), their variability, and their response to different forcing mechanisms. Some studies have estimated air-sea FCO$_{2}$ using in-situ measurements at depths ranging from 1 to 5 m^7–9^ and from 5 to 7 m^10–12^ and below 7 m^13^; these are all considered surface measurements.

Coastal regions and continental/island shelves play important roles in the global carbon cycle. Compared with the global average, carbon fixation ratios are greater in these regions^9,14,15^ due to several factors such as large temperature changes, biological activity, mixing, strong tidal forces, and freshwater inputs (e.g.,^13,16,17^). These factors lead to greater spatial and seasonal variations in surface water pCO$_{2}$ in coastal waters than in open ocean waters. Some authors have estimated FCO$_{2}$ for Atlantic coastal regions; however, the global carbon budget has not fully considered coastal waters due to the reduced number of local and regional studies^18,19^. Warm oceanic wakes are regional phenomena characterized by relatively warm surface waters. This occurs due to the interaction between incoming winds and high mountainous islands, resulting in weaker winds and a clearing of clouds on the leeward side. This leads to intense solar radiation reaching the sea surface, forming a warm oceanic wake. This phenomenon is detectable from space on Madeira Island (northeastern Atlantic Ocean) and can extend 100 km offshore during summer. In this wind-sheltered region, the sea surface temperature can be 4 $^{\circ }$C higher than that of the surrounding oceanic waters (e.g.,^20^). The waters are strongly stratified concerning temperature; the gradient is greater in the first 20 m, creating a daily thermocline^21^. Conversely, the open ocean shows enhanced vertical mixing and greater mixed-layer depth, especially on the island’s southwestern coast^22^ (the exposed region considered in this study).

**Figure 1:**
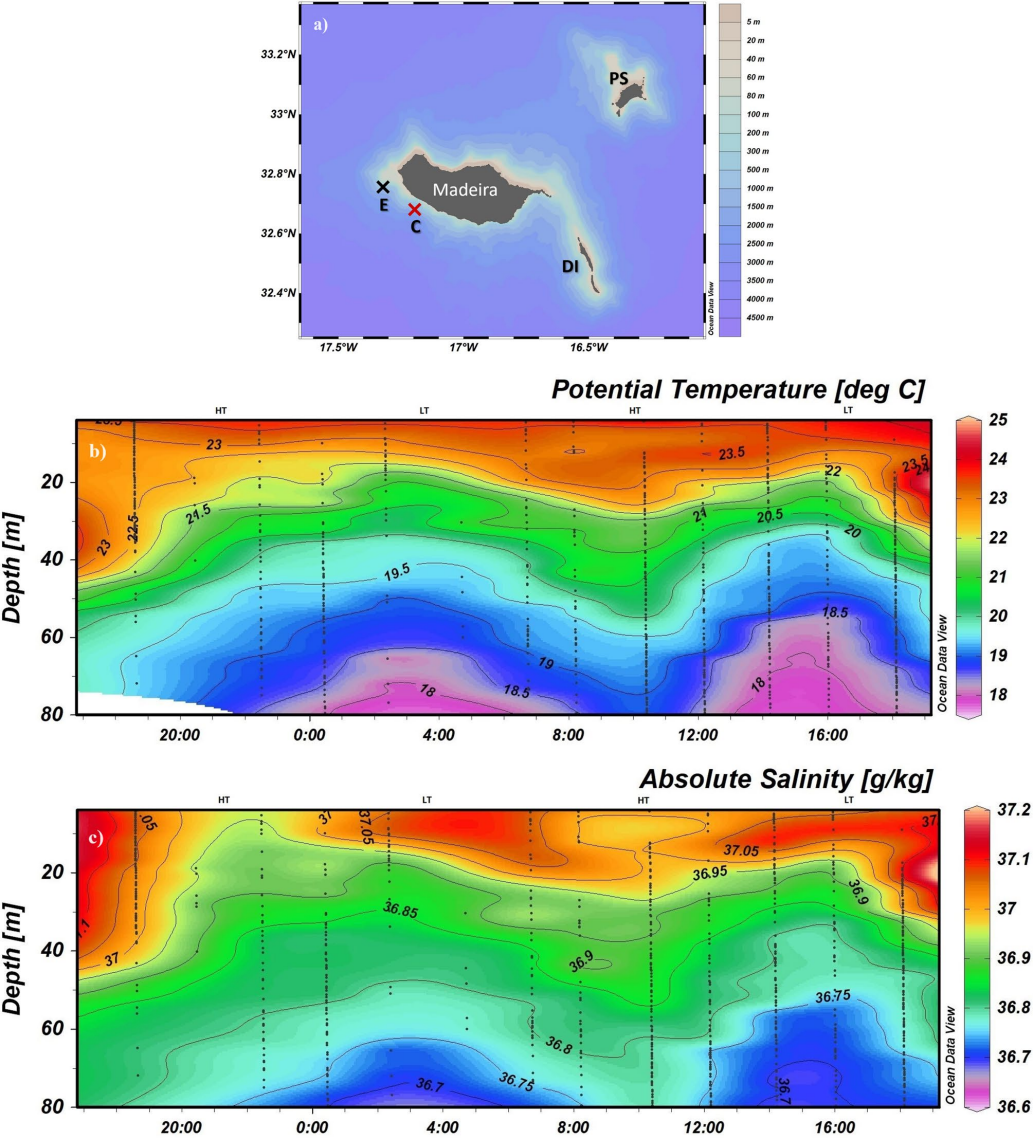
(**a**) Location of the Madeira Archipelago showing Madeira, Porto Santo (PS) and the Desertas Islands (DI), and the sampling stations C (sheltered region, red X) and E (exposed region, black X). HT and LT, at the top of each graphic, indicate high tide and low tide, respectively. (**b**) Potential temperature ( $^{\circ }$C) and (**c**) absolute salinity ($\frac{g}{kg}$) data were collected using the CTD during August 16th at station C (24 h sampling). The data were plotted using the Ocean Data View (ODV5.7.0) software package (https://odv.awi.de/).

The complexity of the processes influencing air-sea exchange and seasonal and spatial variability is among the greatest obstacles to obtaining real values of FCO$_{2}$. In this regard, our study highlights the implications of using partial pressure of CO$_{2}$ (pCO$_{2}$) measurements at different depths in the first layers of the ocean to estimate FCO$_{2}$. In addition, the impact of regional phenomena on the carbon budget is also investigated. To attain our goal, in-situ measurements at different surface depths (1 m, 5 m, and 10 m) were used to analyse the difference in carbon fluxes between a wind-sheltered region and an exposed region. Following the introduction, the paper is organized as follows: “Results” presents the results, including observations. “Discussion” discusses and summarizes the main findings. “Methods” describes the datasets and methods.

## Results

**Figure 2:**
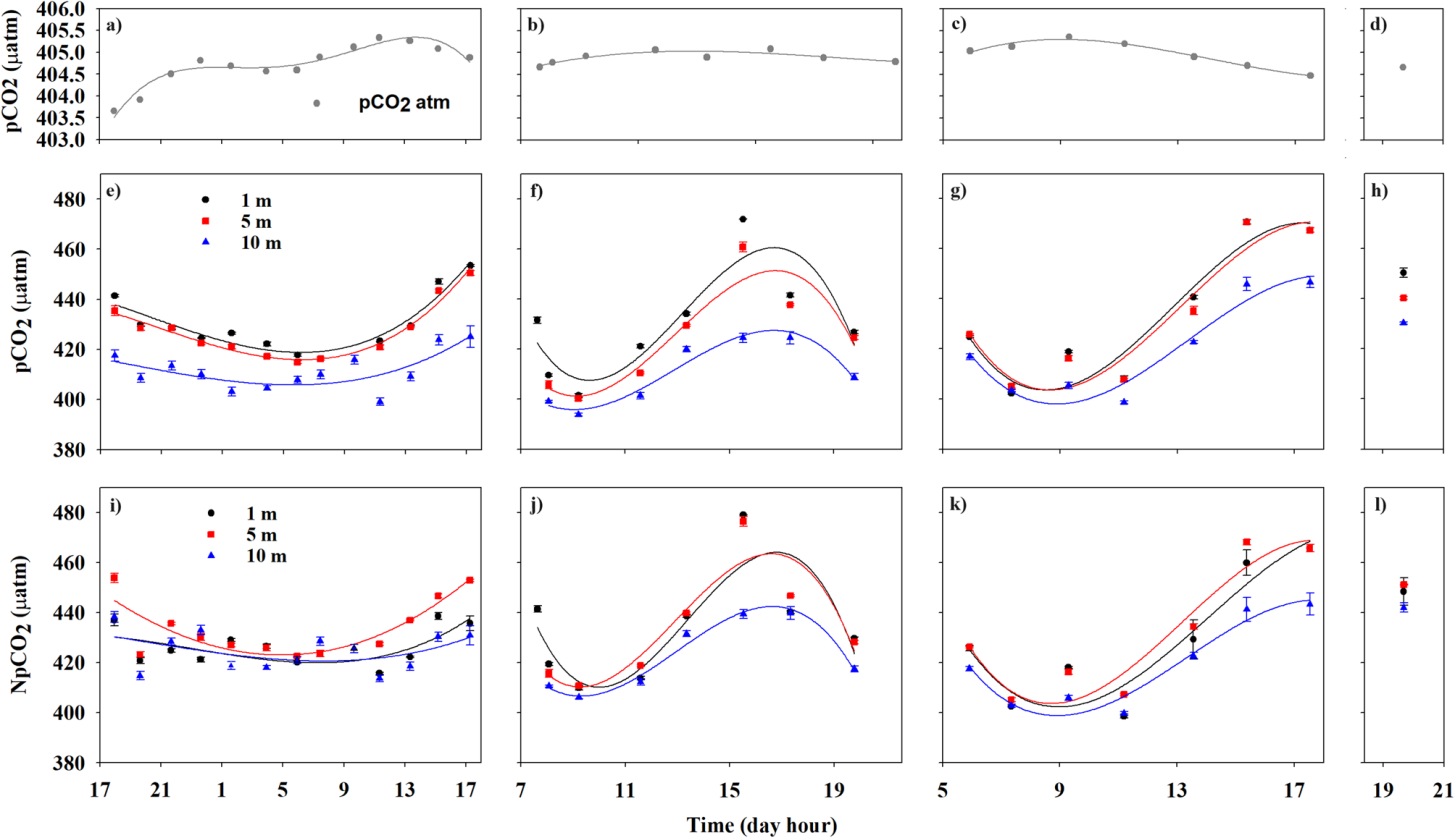
Hourly values of atmospheric pCO$_{2}$ ($\upmu$atm) (**a**–**d**); water pCO$_{2}$ ($\upmu$atm) (**e**–**h**) and NpCO$_{2}$ ($\upmu$atm) (**i**–**l**) at 1 m (black points), 5 m (red squares) and 10 m (blue triangles); in the sheltered region on August 16th (**a**,**e**,**i**), August 20th (**b**,**f**,**j**), and August 23rd (**c**,**g**,**k**) and in the exposed region (**d**,**h**,**l**). The error bars represent the standard deviations between 0–1 m, 5–6 m, and 10–11 m. The lines denote the linear regression (order three) at each depth.

**Figure 3:**
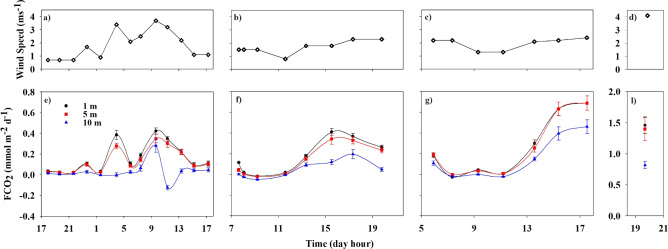
Hourly values of wind speed (ms$^{-1}$; top) and FCO$_{2}$ (mmol m$^{-2}$ day$^{-1}$; bottom) at 1 m (black points), 5 m (red squares) and 10 m (blue triangles) in the sheltered region on August 16th (**a**,**e**), August 20th (**b**,**f**), and August 23rd (**c**,**g**) and in the exposed region (**d**,**h**). The error bars represent the standard deviations between 0–1 m, 5–6 m, and 10–11 m. The lines denote the linear regression (order three) at each depth.

Considering that FCO$_{2}$ varies seasonally and spatially with the water characteristics and wind, this section focuses on the vertical structure of temperature and salinity measured in the wind-sheltered region (Fig. 1); atmospheric and water pCO$_{2}$ and normalized pCO$_{2}$ (NpCO$_{2}$) (Fig. 2); and wind speed (in the lower atmosphere) and calculated FCO$_{2}$ (Fig. 3) in the wind-sheltered and exposed regions. In general, the water column in the wind-sheltered region was stratified, with temperatures being higher at the surface and decreasing with depth (Fig. 1b). The values ranged between 24 and 24.5 $^{\circ }$C at the surface and between 18 and 19 $^{\circ }$C at a depth of 80 m. The impact of solar radiation is noticeable at the first 10 m, with variations occurring only during sunny hours (23 to 24.5 $^{\circ }$C). A distinct influence is perceptible at depths between 10 and 40 m, adding a cycle oscillation at the isotherms in the water column. This oscillation could be related to the tidal cycle. During flood tide (HT, Fig. 1b), the isotherms stretched to greater depths and became more visible during the late afternoon with temperatures of approximately 23.5$^{\circ }$C at a depth of 40 m. During ebb tide (LT), colder waters rise to shallow depths (20 m depth). Below 40 m, the isotherms seem to respond only to the cycle oscillation. The salinity (Fig. 1c) had a similar pattern of variation with temperature throughout the water column. Therefore, salinity gradients were observed instead of a homogeneous layer in the first 10 m. Additionally, a low-salinity water mass at the surface during the flood tide, contrasted with the higher salinity during the ebb tide.

In the wind-sheltered region, the atmospheric pCO$_{2}$ presented daily variations of less than 2 $\upmu$atm (Fig. 2a–c). The values varied by 1.6 $\upmu$atm during sunny hours (between  403.7 $\upmu$atm at 1700 UTC and  405.3 $\upmu$atm at 1120 UTC, the minimum and maximum, respectively, over three days; Fig. 2a–c). Between day and night (Fig. 2a), the values decreased during the night (404.8 $\upmu$atm at 2336 UTC to 404.5 $\upmu$atm at 0555 UTC), increased after sunrise (404.8 $\upmu$atm at 0720 UTC to 405.3 $\upmu$atm at 1120 UTC) and decreased again after the peak heat hour (1300 UTC). The water pCO$_{2}$ varied with sunny hours; in particular, higher values occurred during the day (0800 UTC and 1700 UTC; Fig. 2e), when higher temperatures were recorded (Fig. 1a). This variation is visible at all depths and on all three days (Fig. 2e–g), with less amplitude at a depth of 10 m (blue line in Fig. 2). The water pCO$_{2}$ values ranged from approximately 406 to 465 $\upmu$atm at 1 m, from 407 to 460 $\upmu$atm at 5 m and from 396 to 430 $\upmu$atm at 10 m (at 0800 UTC and 1700 UTC, respectively). The discrepancy in the depths from 1 to 5 m is lower (approximately 5 $\upmu$atm; 1%) than that from 5 to 10 m (approximately 20 $\upmu$atm; 4%). In the exposed region (Fig. 2h), this discrepancy is identical among the three depths, at 2.29% and 1.97% for the 1 to 5 m and 5 to 10 m depths, respectively.

After normalizing the water pCO$_{2}$ to a constant temperature of 24 $^{\circ }$C (Fig. 2i–l) to account for the contribution of physical and biological processes to the observed variability, the activity of NpCO$_{2}$ throughout the day was maintained, but in general, the values increased at depths of 5 and 10 m. This occurred in both regions, i.e., the sheltered and exposed regions. At 5 m depth (red line), the NpCO$_{2}$ values were equal to or even greater than those at 1 m depth (black line) on all three days. The values at 10 m depth (blue line) also changed and were more elevated; moreover, on August 16th, the NpCO$_{2}$ values at 10 m were similar to those at 1 m depth. The discrepancy among the three depths decreased in both regions, with less variation in the NpCO$_{2}$ values in the exposed region.

The calculated fluxes (Fig. 3) were greater in the exposed region (maximum of 1.46 mmol m$^{-2}$ day$^{-1}$; Fig. 3h) than in the sheltered region (maximum of 0.69 mmol m$^{-2}$ day$^{-1}$; Fig. 3e–g), at all depths. In general, in the sheltered region, the FCO$_{2}$ behaviour was consistent with that of pCO$_{2}$; i.e., higher values of pCO$_{2}$ indicate greater fluxes (Fig. 3f,g). However, on August 16th (Fig. 3e), FCO$_{2}$ showed significant variations related to wind variability (Fig. 3a–d). Positive peaks in FCO$_{2}$ occurred in response to wind speed intensification (e.g., 0.1 mmol m$^{-2}$ day$^{-1}$ at 1.7 ms$^{-1}$ at 0000 UTC; 0.39 mmol m$^{-2}$ day$^{-1}$ at 3.4 ms$^{-1}$ at 0400 UTC). In contrast, when the wind speed decreased, the FCO$_{2}$ also decreased (e.g., 0.03 mmol m$^{-2}$ day$^{-1}$ at 0.9 ms$^{-1}$ at 0130 UTC; 0.11 mmol m$^{-2}$ day$^{-1}$ at 2.1 ms$^{-1}$ at 0600 UTC). These peaks occurred with lower pCO$_{2}$ during the nighttime at depths of 1 and 5 m. After sunrise (0900 UTC), another FCO$_{2}$ peak was observed (0.42 mmol m$^{-2}$ day$^{-1}$) with a higher wind speed (3.7 ms$^{-1}$) and an increase in pCO$_{2}$ (423 $\upmu$atm; Fig. 2a). At the 10 m depth the FCO$_{2}$ was close to zero, except during the morning (0725–1130 UTC), when it increased, followed by a decrease in pCO$_{2}$. This is the only time that the ocean behaved as a sink for atmospheric CO$_{2}$. Comparison of the FCO$_{2}$ (calculated with pCO$_2$ measurements) in both regions at the same time revealed that the values decreased by approximately 12% in the sheltered region (0.26–0.23 mmol m$^{-2}$ day$^{-1}$) and 6% in the exposed region (1.46 to 1.38 mmol m$^{-2}$ day$^{-1}$), at depths from 1 to 5 m. However, considering the higher peaks of FCO$_{2}$ in the sheltered region (0.39 to 0.28 mmol m$^{-2}$ day$^{-1}$), the decrease could reach 28% at depths between 1 and 5 m and 99% between 1 and 10 m. In the exposed region at 10 m, the decrease reached 44% compared with that at 1 m depth.

## Discussion

A precise assessment of spatial and seasonal variability^8^ and a greater characterization of coastal regions^14^ are fundamental for improving our knowledge of the impacts of oceanographic and meteorological processes on the carbon cycle.

To date, most scientific studies have focused on using shipboard CO$_{2}$ measurements to calculate air-sea fluxes (i.e., FCO$_{2}$) directly^11–13,23^, extrapolating the values in time and space^8^, or even using parameterizations based on surface water properties^10^. Although the data contain original CO$_{2}$ surface water measurements, such measurements are usually made several meters below the surface, which can be a source of potential error in FCO$_{2}$ calculations^6,24^. This potential error arises from the assumption of vertical homogeneity within the mixed layer^25^. Therefore, if vertical concentration gradients exist in the mixed layer, as is the case in the wind-sheltered region with stratified temperature and salinity layers (Fig. 1a,b), then underway seawater is not representative of the surface boundary layer, which could create a global sampling bias^26^.

Despite pronounced seasonal variations^17^, the North Atlantic has been recognized as one of the largest ocean sinks of CO$_{2}$, especially at subtropical latitudes (e.g.,^8,27–29^)^23^, studied the seasonal variability in CO$_{2}$ in the Northeast Atlantic Ocean between the northwestern African coast and the open-ocean waters of the North Atlantic subtropical gyre. The results showed that during 2019, the region behaved as an annual CO$_{2}$ sink of -2.65 ± 0.44 Tg CO$_{2}$ year$^{-1}$. However, during the warm months, this entire region acted as a CO$_{2}$ source^23,30,31^. In our study, which was performed in summer, the sheltered and exposed regions acted predominantly as a source of CO$_{2}$, in agreement with the literature. The higher values of pCO$_{2}$ during sunny hours (in the wind-sheltered region, Fig. 2a–c) are consistent with the highest sea surface temperature (Fig. 1a) and vice versa. This result aligns with the study by^26^, which investigated the influence of solar heat-trapping and near-surface warming on CO$_{2}$ gas exchange. The study noted that the existence of a warm oceanic surface layer creates a net asymmetry in CO$_{2}$ transfer between the ocean and atmosphere. The warming and cooling cycle of the upper ocean leads to a decrease in CO$_{2}$ invasion and an increase in CO$_{2}$ evasion, impacting the net daily exchange of CO$_{2}$, which is consistent with our findings (in the wind-sheltered region; Figs. 1 and  2). Similarly^32^, reported that in subtropical waters, CO$_{2}$ transfer conditions were governed primarily by temperature. According to our results, after normalizing the pCO$_{2}$ to a constant temperature of 24 $^{\circ }$C an increase in the values was observed (Fig. 2e–h) principally in the deepest layers. This should be ascribed to vertical mixing processes driven by tidal effects, bringing up the coldest and remineralized deeper waters, as happens on the salinity of the deepest layers (Fig. 1b). On the other hand, at night, the pCO$_{2}$ concentrations decreased, and concurrently with higher wind speeds, the FCO$_{2}$ increased (see 0400 UTC; Fig. 3a), indicating that CO$_{2}$ was transferred to the atmosphere. Reference^33^ affirms that higher winds to a static $\triangle pCO_{2}$ (without thermodynamic forces) can act synergistically on the trend in FCO$_{2}$. Although not investigated in this work, thermal and haline skin effects affect the FCO$_{2}$ calculation. These effects should be considered in more complete future research. Despite being only a few millimeters thick at the sea surface and generally weaker than the thermal effects^6^, the thermal skin effect increases oceanic global uptake^25^. Additionally, according to^6^, the salty skin effect accounts for approximately one-sixth of the thermal effect. Nevertheless, it is also important to note the difference in FCO$_{2}$ between the study regions. Although both acted as sources, FCO$_{2}$ varied from 1.46 mmol $m^{-2}d^{-1}$ in the exposed region to 0.26 mmol $m^{-2}d^{-1}$ in the sheltered region, corresponding to 82% of the difference at the same time. Some global ocean-atmosphere FCO$_{2}$ studies have excluded coastal regions^8,10,11^. Recently, the Integrated Ocean Carbon Research report^34^ affirmed that coastal and marginal seas remain understudied. Laruelle et al.^18^ reported that CO$_{2}$ fluxes could become 40% more intense in ice-free surface regions than in exposed regions. In^14^, it was determined that the inclusion of coastal zones increased the estimated global ocean CO$_{2}$ sinks by 57% at high latitudes and by 15% at mid-latitudes, while CO$_{2}$ emissions from the ocean to the atmosphere increased by 13% in tropical and subtropical regions.

**Figure 4:**
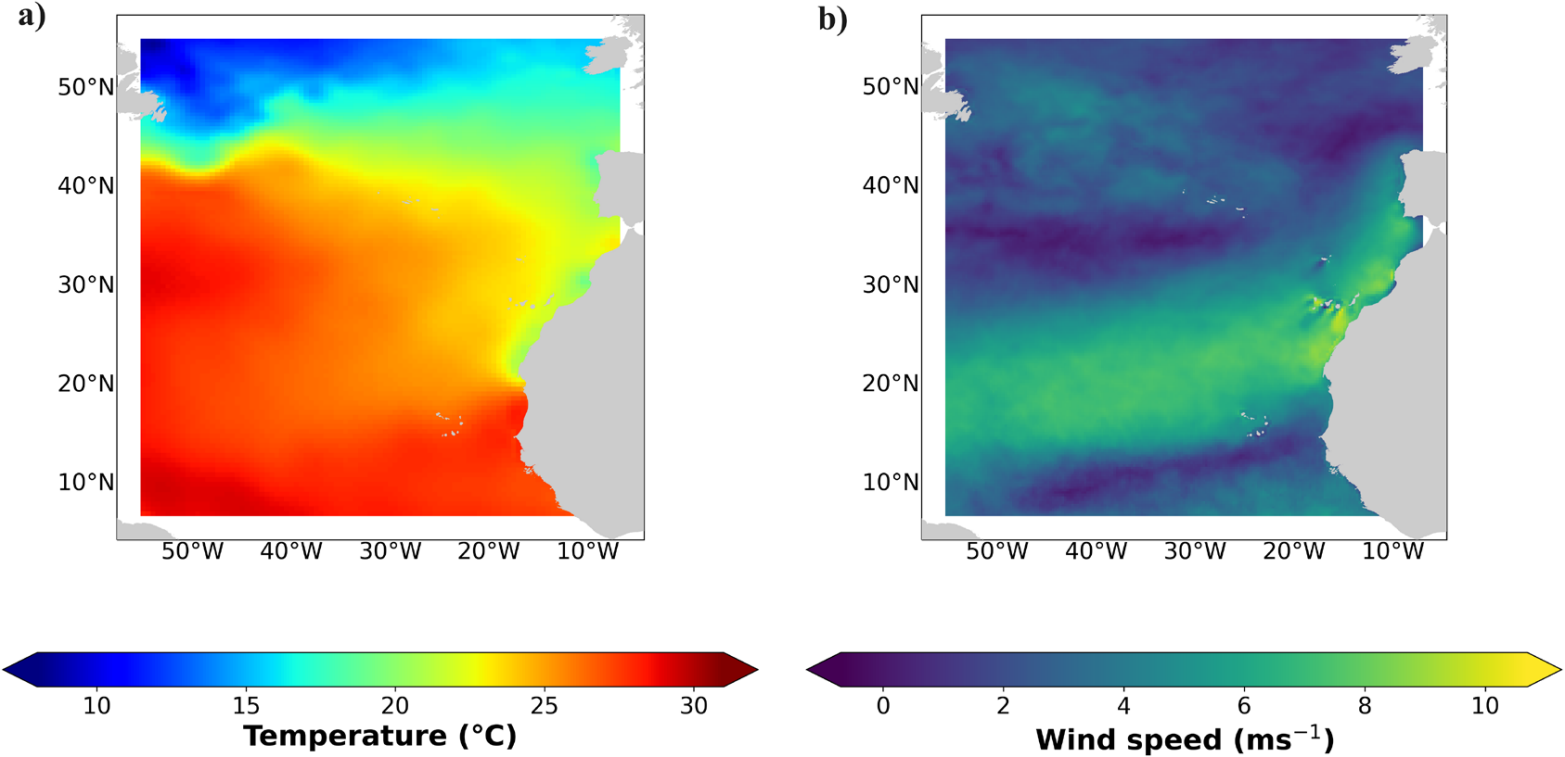
Mean sea surface (**a**) temperature ($^{\circ }$C) from the CMEMS in-situ near real-time database and (**b**) wind speed (ms$^{-1}$) from CMEMS scatterometer data and model for August in the Atlantic Basin. The data were plotted using a mapping package for Python (https://www.python.org/).

**Figure 5:**
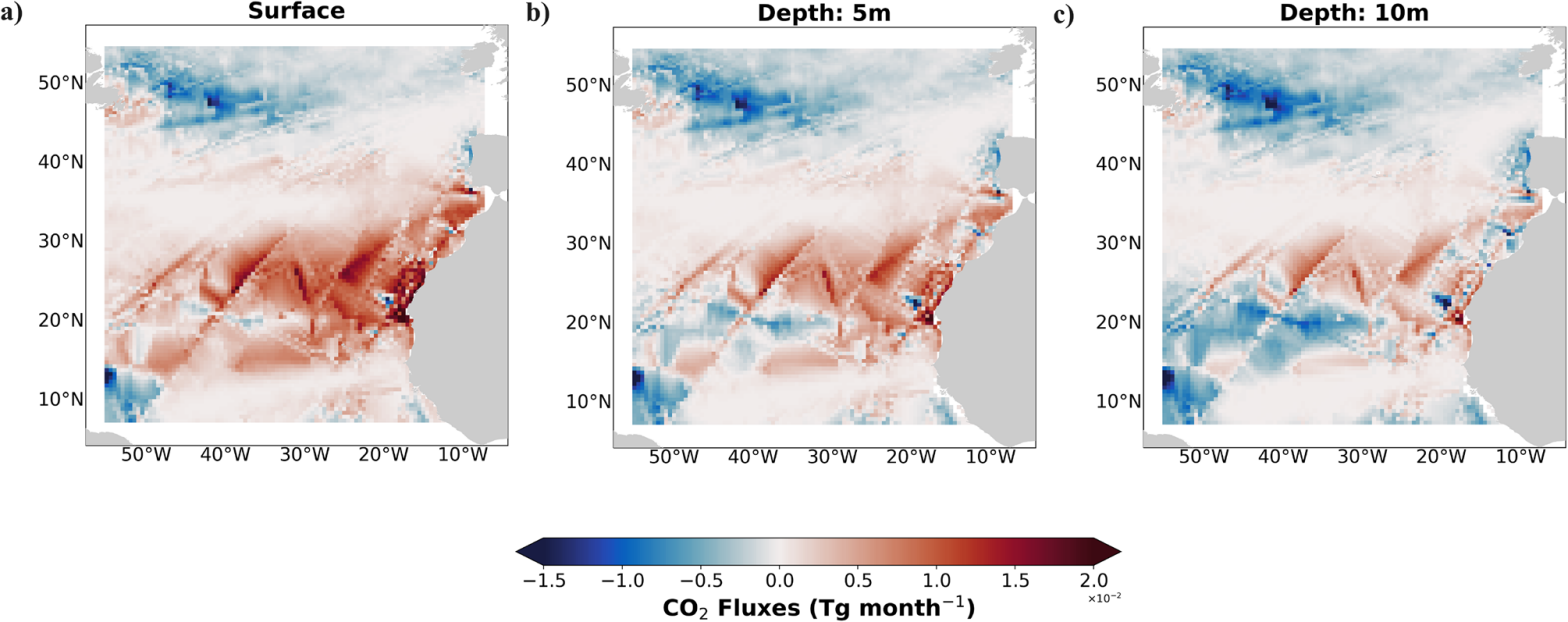
August mean CO$_{2}$ fluxes (Tg month$^{-1}$; by SOCATv2022^38^) estimated for the Atlantic basin at the (**a**) surface (**b**) 5 m depth and (**c**) 10 m depth. The data were plotted using a mapping package for Python (https://www.python.org/).

Our results showed the underestimated impact of using pCO$_{2}$ measurements at different ocean depths on FCO$_{2}$ estimation at the local scale. However, what impact could such underestimation have on the Atlantic Ocean basin? In this sense, the FCO$_{2}$ was calculated for the Atlantic North Basin using the underestimation values obtained in this study at the exposed region (2.29% and 1.97% for the 1 m and 10 m depths, respectively). Figure 4 shows the sea surface temperature (Fig. 4a) and the wind speed (Fig. 4b) in the Atlantic North Basin. According to this figure, the temperature of the sea surface (Fig. 4a) increased from the north ($\sim$ 12 $^{\circ }$C; mid-latitudes) to the south ($\sim$ 27 $^{\circ }$C; tropics). On the African coast and surrounding the archipelagos of Madeira and the Canary Islands, the values were lower (between 20 and 24 $^{\circ }$C) than those at the same latitude (27 $^{\circ }$C; e.g., 32$^{\circ }$ N). The wind speed (Fig. 4b), on the other hand, had greater values (11 ms$^{-1}$) close to these regions. In turn, the weakest winds (until 2 ms$^{-1}$ ) can be observed in the northeast, close to the Azores and south of the Cape Verde archipelagos. The FCO$_{2}$ estimates at depths of 1, 5, and 10 m are shown in Fig. 5a–c, respectively. Throughout August, at the mid-latitudes, the ocean acted as a CO$_{2}$ sink (blue in Fig. 5), while in the tropics, the ocean acted as a source (red in Fig. 5) of CO$_{2}$. The fluxes were almost zero in the subtropics; despite the higher temperatures reported in this region (in the range of 24–26 $^{\circ }$C), the winds were weaker. Here, the African coast and surrounding archipelagos of Madeira and the Canary Islands were exceptions; the orographic winds exhibited greater values, between $\sim$ 9 and 11 ms$^{-1}$, and although the sea surface temperatures ranged from 22 to 24 $^{\circ }$C, the FCO$_{2}$ displayed higher values for the Atlantic basin. The lowest sea surface temperature, between 15 and 17 $^{\circ }$C, and the stronger winds ($\sim$ 9 ms$^{-1}$) in the northwestern part of the mid-latitudes created an intense CO$_{2}$ sink region in the ocean. Considering the depths of the measurements, the FCO$_{2}$ values changed significantly between the surface and depths of 5 and 10 m. Although the pattern persisted, the values decreased substantially from the surface to a depth of 10 m. In the latter scenario, some regions of the tropical ocean transitioned from source to sink. According to the SOCAT dataset, at a depth of 5 m, the Atlantic basin emits approximately 0.29 Tg month$^{-1}$ of CO$_{2}$ to the atmosphere. The estimated FCO$_{2}$ (with pCO$_2$ measurements) at 1 m depth, 12.02 Tg month$^{-1}$, is 97.6% greater than that estimated at 5 m depth, despite the minor difference (2.29%) in pCO$_2$, which was found in our study at the same depths. Observation of the FCO$_{2}$ estimated with the pCO$_2$ at 10 m revealed that this behaviour reversed, and the FCO$_{2}$ in the Atlantic Basin decreased to 9.85 Tg month$^{-1}$. FCO$_{2}$ values change significantly with the depths of in-situ measurements, indicating the significance of proper measurement acquisition. More studies should be performed to confirm the trends in different regions, and modelling studies should consider this important variability in flux calculations. Future studies should also continue to evaluate differences in pCO$_2$ at different depths.

## Methods

Identifying and quantifying near-surface gradients in trace gas concentrations is challenging. Several instruments were used to capture the response of the ocean’s surface layer to atmospheric forcing. Data acquisition was performed during a summer campaign in 2021 (16–23 August) onboard a vessel of opportunity. On August 16 (24 h), 20 (12 h) and 23 (12 h), oceanographic and meteorological data were collected every 2 h at station C (Fig. 1); at station E, data were collected on August 23. In the ocean, temperature and salinity were measured, while air temperature and wind speed were measured in the lower atmosphere. pCO$_{2}$ was recorded in the ocean and lower-atmosphere.

In the ocean, data were acquired using a conductivity, temperature, and depth profiler (pumped CTD, SeaBird-19). The CTD was measured at 4 Hz, and a total of 13 vertical profiles were obtained. The acquisition was carried out using Seaterm software, and the processing included a set of SBE Data Processing routines (Sea-Bird Electronics), as detailed in^21^.

Furthermore, the partial pressure of CO$_{2}$ gas dissolved in water was measured with a pCO$_{2}$ sensor (submersible sensor, Pro Oceanus) using infrared detection at a sample rate of 1 second and a resolution of 0.01 ppm. For a consistent concentration, the sensor was preequilibrated under seawater conditions for a 30-min period. To achieve equilibrium between the pCO$_{2}$ membrane and the seawater at depth, the sensor was lowered at a rate of 3 m/min. The data were classified into three depths using the mean: 0–1 m, 5–6 m, and 10–11 m. The standard deviation was calculated. pCO$_{2}$ was also measured in the lower atmosphere by pumping air instead of seawater. An SBE-37 sensor, used to record temperature, salinity, and pressure, was combined with the pCO$_{2}$ sensor, and samples were processed at a sampling rate of 1 second.

Air-sea fluxes of carbon dioxide are commonly determined by first measuring partial pressure gradients between the ocean surface and lower atmosphere and then multiplying them by a parameter called the gas transfer velocity. The CO$_{2}$ fluxes were determined using Eq. (1):

1 $$\begin{aligned} FCO_{2} = 0.24 S k \triangle pCO_{2} \end{aligned}$$

where 0.24 is a conversion factor to express data in mmol m$^{-2}$ day$^{-1}$; S is the solubility of CO$_{2}$ in seawater; and $\bigtriangleup pCO_{2}$ is the difference between the seawater and low atmosphere (pCO$_{2,sw}$ - pCO$_{2,atm}$). Positive fluxes indicate that the ocean acts as a source of CO$_{2}$ to the atmosphere, while negative fluxes indicate that the ocean acts as an atmospheric CO$_{2}$ sink.

The gas transfer velocity is usually parameterized as a function of the wind speed. Wind does not directly control gas transfer; more precisely, gas transfer is governed by complex boundary layer processes. However, most of these boundary layer processes are strongly influenced by wind, and on a global scale, wind can be used as the sole environmental forcing. The parameterization of^35^ was used in this study, with k (cm h$^{-1}$) being the gas transfer rate expressed in Eq. (2):

2 $$\begin{aligned} k = 0.251 <U^2> \left( \frac{Sc}{660} \right) ^{-0.5} \end{aligned}$$

where U is the wind speed (ms$^-1$) and Sc is the Schmidt number (kinematic viscosity of seawater) divided by the gas diffusion coefficient. This parameterization contributes to the uncertainty of the flux. The relationship between wind speed and gas exchange was studied by^35^, and the uncertainty reached 20% for a basin-scale application. Woolf et al.^36^ also analysed this uncertainty using calculations referenced to 2010 and concluded that a realistic estimate is approximately 9%. Woolf et al.^36^ also referred to temperature gradients as a source of uncertainty. To remove the thermal effect on daily variation, pCO$_{2}$ was normalized to a constant temperature of 24 $^{\circ }$C using a mean coefficient of 0.0423 $^{\circ }$C$^{-1}$, determined experimentally by^32^ (and confirmed by^37^) for a North Atlantic surface water sample and using Eq. 3:

3 $$\begin{aligned} NpCO_{2} = (pCO_{2})_{obs} \times exp[ 0.0423 (24-T_{obs})] \end{aligned}$$

In the atmosphere, vertical profiles were determined via atmospheric radiosondes (DFM-09, GRAW Radiosondes). The atmospheric radiosondes measured profiles of air temperature (accuracy < 0.2 $^{\circ }$C), air pressure (accuracy < 0.3 hPa), wind speed (accuracy < 0.2 ms$^-1$) and wind direction. All the sensors were calibrated.

The FCO$_{2}$ was estimated for the North Atlantic Basin using sea surface pCO$_{2}$ from the Surface Ocean CO_2_ Atlas (SOCAT v2022^38^), global ocean monthly temperature and salinity (in-situ measurements^39^), global ocean monthly mean sea surface wind from Copernicus (scatterometer data and model^40^) and global hourly surface pressure data (reanalysis^41^). The details of the data processing are presented below. The SOCAT pCO$_{2}$ measurements span 1963 through 2021. These types of measurements are often collected from the underway seawater intake of research vessels for a depth range of 2–7 m (our study treats this range of depths as 5 m). To adjust the pCO$_{2}$ values for 2021, an increment of 1.7 $\upmu$atm/year was computed using the monthly mean carbon dioxide data from the Mauna Loa Observatory, Hawaii (https://gml.noaa.gov/ccgg/trends/data.html). Using the actualized pCO$_{2}$ values, a linear interpolation was carried out throughout the entire North Atlantic Ocean using the SST grid, which has a spatial resolution of 0.5$^{\circ }$. Afterwards, the underestimation values obtained in this study (2.29% and 1.97% for 1 m and 10 m depths, respectively) were assumed to be valid for the North Atlantic and applied to the interpolated pCO$_{2}$ values, considering only August. Finally, the FCO$_{2}$ was calculated using Eq. (1) and the wind product.

## Data Availability

The datasets used and/or analysed during the current study are available from the corresponding author upon reasonable request.
